# Toward Targeted Kinetic Trapping of Organic–Inorganic Interfaces: A Computational Case Study

**DOI:** 10.1021/acsphyschemau.1c00015

**Published:** 2021-10-11

**Authors:** Anna Werkovits, Andreas Jeindl, Lukas Hörmann, Johannes J. Cartus, Oliver T. Hofmann

**Affiliations:** Institute of Solid State Physics, TU Graz, NAWI Graz, Petersgasse 16/II, 8010 Graz, Austria

**Keywords:** thin film growth, diffusion, reorientation, transition rates, density functional theory, nudged elastic band method, transition state theory, phase transition

## Abstract

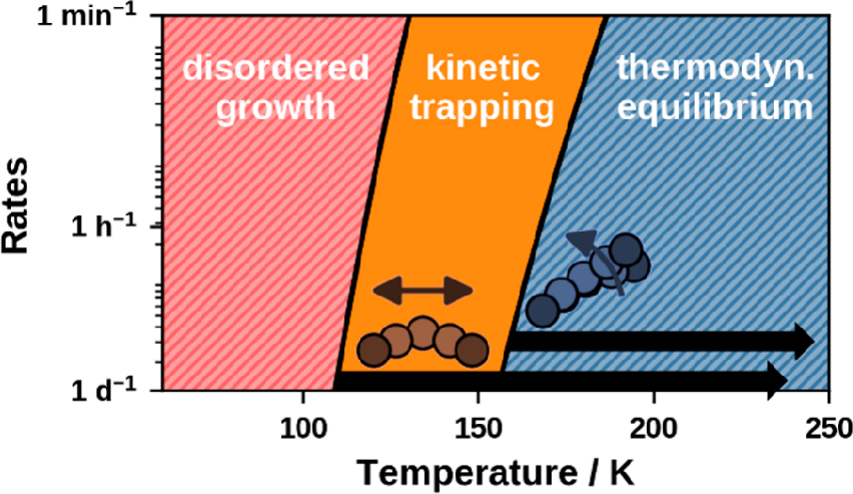

Properties of inorganic–organic interfaces, such as their interface dipole, strongly depend on the structural arrangements of the organic molecules. A prime example is tetracyanoethylene (TCNE) on Cu(111), which shows two different phases with significantly different work functions. However, the thermodynamically preferred phase is not always the one that is best suited for a given application. Rather, it may be desirable to selectively grow a kinetically trapped structure. In this work, we employ density functional theory and transition state theory to discuss under which conditions such a kinetic trapping might be possible for the model system of TCNE on Cu. Specifically, we want to trap the molecules in the first layer in a flat-lying orientation. This requires temperatures that are sufficiently low to suppress the reorientation of the molecules, which is thermodynamically more favorable for high dosages, but still high enough to enable ordered growth through diffusion of molecules. On the basis of the temperature-dependent diffusion and reorientation rates, we propose a temperature range at which the reorientation can be successfully suppressed.

## 1 Introduction

Metal–organic interfaces act as a basis for a variety of possible nanotechnological applications, such as molecular switches,^1,2^ thermoelectrics,^3,4^ memories,^5^ transistors,^6−8^ or spintronic devices.^9^ Owing to the advances in computational material design, possibilities for developing functional interfaces with tailored physical properties and functionalities have increased in the last decades.^10,11^ However, the functionality of these interfaces does not depend on the choice of the metal and the organic component alone. Rather, also the structure the organic component assumes on the surface plays a decisive role. A prime example are molecular acceptors that undergo a (coverage-dependent) reorientation from flat-lying to upright-standing positions, such as hexaazatriphenylene-hexacarbonitrile (HATCN) and dinitropyrene-tetraone (NO_2_-Pyt) on Ag(111).^12,13^ Because the electron affinity of organic films depends on their orientation,^14^ this is accompanied by significant changes of the charge transfer and interface work functions.^13,15^ In the two examples above, the structural transition causes a change of the work function of more than 1 eV, illustrating how important control over the structure is.

In principle, such control can be achieved by identifying process conditions that allow the target structure to grow in thermodynamic equilibrium.^16,17^ In practice, however, often kinetically trapped phases appear, especially when preparing interfaces using physical vapor deposition.^18^ This is because kinetics plays a major role: Following Ostwald’s rule of stages,^19^ thermodynamically less stable structures form first. Whether the transition to a more stable structure occurs or whether the structure becomes kinetically trapped depends on the energetic barriers and the corresponding transition rates. Therefore, we can make a virtue out of a necessity by explicitly utilizing kinetic trapping to grow structures out of thermodynamical equilibrium: In theory, controlled formation of a kinetically trapped structure should be possible by selecting a deposition temperature at which the rate for the phase transition to a thermodynamically more stable structure is slower than the speed at which the trapped structure grows. This requires profound knowledge of (a) the underlying transition mechanisms, and (b) the ability of the molecules to diffuse and aggregate, that is, to form a seed for a different structure vis-à-vis to continue growing in the less thermodynamically stable form.

In this work, we perform a first step to predict controlled growth of the model system tetracyanoethylene (TCNE) on Cu(111). While being computationally more tractable than its cousins HATCN and NO_2_–PyT on Ag(111), it reveals an even larger change in work function. When increasing the dosage of TCNE, the system undergoes a reorientation from flat-lying to upright-standing molecules,^20^ which leads to a work function increase of approximately 3 eV. When continuing growth, a second layer of TCNE forms on top of the first, standing, monolayer.^20^

As the layer in direct contact with the surface is the decisive factor for the properties of the interface,^7^ it is highly interesting to study how the reorientation within the first layer could be suppressed for high dosages. To take a first step in predicting how the reorientation of TCNE on Cu(111) could be prevented, here we study, by first-principles, under which conditions the reorientation can be kinetically suppressed altogether already for individual TCNE molecules, that is, when not even a single molecule is able to adopt the upright-standing geometry within a reasonable time scale. However, computing TCNE on Cu(111) faces a fundamental challenge: The reorientation on the surface substantially alters the way the molecules interact with the surface.^20^ This includes charge transfer and the connected rehybridization of molecular and metal orbitals. These orbital rehybridizations are not covered by state-of-the-art force-field approaches rendering them (and, by extension, molecular dynamics simulations) inapplicable here. Instead, we use dispersion-corrected density functional theory (for details see Methods) to obtain minimum energy paths and transition states by the nudged elastic band method.^21,22^ This method was previously successfully employed to study diffusion processes of inorganic–organic interfaces.^23,24^ Applying harmonic transition state theory,^25,26^ we can further determine temperature-dependent rates of diffusion and reorientation. This allows us to estimate a temperature range at which the reorientation is suppressed while further growth of lying seeds is still supported, resulting in a kinetic trapping of lying TCNE.

## 2 Results and Discussion

### 2.1 Transition Paths and Barriers

Arguably, the ability of the system to undergo a phase transition depends on the molecules’ ability to diffuse on the surface and, more importantly, on the rate at which they can change their orientation. Generally, reorientation processes happen spontaneously and are typically energetically driven: Once a sufficient number of molecules aggregate in the upright-standing geometry (i.e., they exceed a so-called critical nucleus size), this geometry becomes energetically favorable compared to flat-lying geometries. The number of molecules for this critical cluster size varies from system to system (and is thought to be between 3 and 10).^27−30^ However, collaborative reorientations notwithstanding, it is clear that at least a single molecule must reorient (i.e., some molecule has to make the first step). This provides a limit to the rate at which critical clusters can form in the first place.

Consequently, a useful first step is to investigate these processes for individual molecules, rather than directly studying transitions between full close-packed structures. In our case, this is justified because both, the most favorable flat-lying and the most favorable upright-standing structure, consist of molecular geometries that would also be stable local minima on their own due to the strong molecule–substrate interactions. In addition, this reduced complexity enables studying kinetic processes at a feasible cost. Therefore, we omit multimolecule processes that include intermolecular interactions, such as the initial nucleation, attachment and detachment processes from an island, and Ehrlich-Schwoebl barriers. Instead, we focus exclusively on two fundamental aspects in the low coverage growth regime: The diffusion and the reorientation of individual molecules on the surface.

Before we explain which transitions we compute in detail, we briefly introduce the stable adsorption geometries of individual TCNE molecules on Cu(111) and the two structures in which we are interested. This information was previously provided by Egger et al.^20^ and is repeated here, as the local geometries are the starting points for all our further computations.

**Figure 1:**
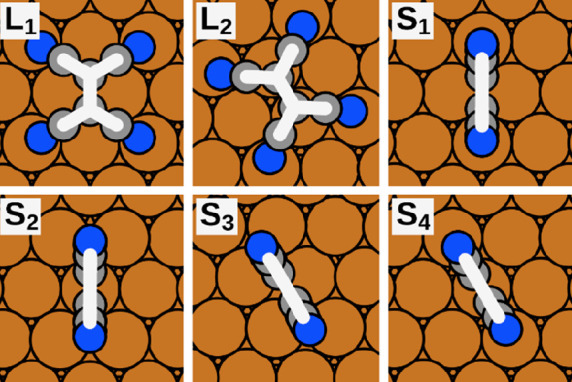
Top view of stable adsorption geometries for TCNE on Cu(111).^20^ The orange spheres represent the Cu atoms of the substrate, whereas the gray and blue spheres are the C and N atoms, respectively. The white overlay is a reduced representation used in further plots.

The most favorable way for individual TCNE molecules to adsorb on the Cu(111) surface is in a “flat-lying” position. For this case, there are two different possible adsorption geometries (i.e., local minima) for the molecule, which we will further denote as **L**_**1**_ and **L**_**2**_ (see Figure 1). There are also four “upright-standing” adsorption geometries, denoted as **S**_**1**_ to **S**_**4**_ (also shown in Figure 1). Their energies are more than 0.5 eV higher, that is, less stable.

In a full monolayer, at low coverages the energetically most favorable structure consists exclusively of flat-lying molecules in the **L**_**1**_ position, while its pendant for high coverages includes the upright-standing positions **S**_**1**_, **S**_**3**_, and **S**_**4**_ (for details see Supporting Information and [^20^]). It is clear that for the flat-lying structure to grow, lying molecules must be able to diffuse on the surface (“lying diffusion”). The formation of the upright-standing structure requires that molecules can reorient from lying to standing (“reorientation”), and, potentially, that the standing molecules can also diffuse on the surface (“standing diffusion”).

**Figure 2:**
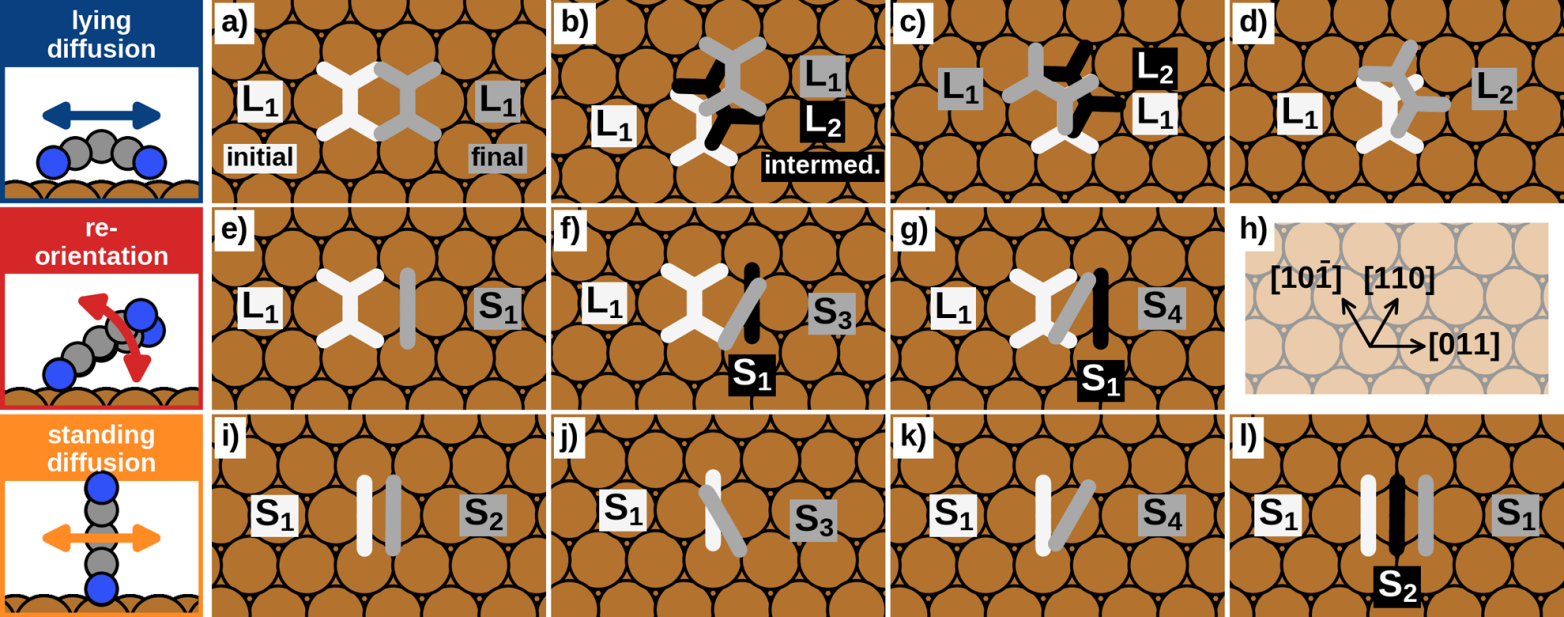
Selected start- (white) and end points (gray) of the transitions for the lying diffusion (a–d), reorientation (e–g) and the standing diffusion (i–l). Intermediate steps of multistep transitions (nonelementary transitions) are colored black. For a clear representation, the molecule geometries are displayed in a reduced form that omits the nitrogen atoms, corresponding to the overlay in Figure 1. In panel h, directions of the substrate lattice are stated.

To study diffusion and reorientations, we create a representative set of distinct transitions between pairs of adsorption geometries (including their rotational and translational symmetry equivalents). When naïvely accounting only for, three rotational and three translational symmetry equivalents for each adsorption geometry, we would already get  transitions. Nevertheless, these will decompose into a manageable set of symmetry equivalent “elementary” transitions, which are transitions that possess exactly one transition state and therefore proceed in a single step. Knowing these elementary transitions, pathways can be constructed by linking individual elementary paths in a way that yields the lowest energetic barrier for the total transition. To efficiently obtain the most relevant elementary transitions, sets of start- and end points are selected based on two concepts: First, we restrict the selection to adsorption geometries in adjacent adsorption sites (i.e., translationally equivalent adsorption positions which are anchored on neighboring Cu atoms on the surface), implying that the adsorbate centers are at maximum one Cu-lattice constant apart. In other words, we neglect so-called “long jumps”. This is warranted because there is evidence that such long jumps are improbable for moderate to low temperatures and for small molecules.^31,32^ Second, we assume that for moderate to low temperatures kinetics is mainly dominated by transitions including the adsorption geometry with the lowest energy in its class either as start and/or end point (i.e., geometry **L**_**1**_ for flat-lying adsorbates and **S**_**1**_ for the upright-standing ones). This is warranted because low-energy structures also tend to have low-energy barriers due to their wide basin of attraction.^33−36^ This assumption is also confirmed in hindsight by our results (*vide infra*). Therefore, we initialized 10 transitions as depicted in Figure 2a–j. Although this does not provide all possible transitions, with this strategy we expect to obtain the most dominant and thus limiting processes of the distinct transition regimes. To conveniently indicate transitions from adsorption geometry **A** to adsorption geometry **B**, we use a notation of the form **A** → **B**. Hereby, **A** → **B** is simply referred to as “forward” transition, while the transition with inverted initial and final states (**B** → **A**) is denoted as “reverse” transition.

To model the diffusion of lying TCNE molecules, we consider four possible transitions: Three different transitions that go directly from one **L**_**1**_ to another **L**_**1**_ at a different adsorption site (Figure 2a–c), and the transition from **L**_**1**_ to the nearest **L**_**2**_ geometry (Figure 2d). The different **L**_**1**_ → **L**_**1**_ transitions consist of two direct transitions to neighboring adsorption sites (shown in Figure 2a,b) and one transition to a symmetry-equivalent rotated geometry (Figure 2c).

For the reorientation, we consider transitions from the lying minimum **L**_**1**_ to the standing end points **S**_**1**_ (Figure 2e), **S**_**3**_ (Figure 2f) and **S**_**4**_ (Figure 2g), that is, to each of the geometries contained in the upright-standing structure.

For the diffusion of standing molecules, the most favorable adsorption geometry of this class, **S**_**1**_, is always chosen as initial minimum that transitions into either **S**_**1**_ (Figure 2l), **S**_**2**_ (Figure 2i), **S**_**3**_ (Figure 2j) or **S**_**4**_ (Figure 2k).

**Figure 3:**
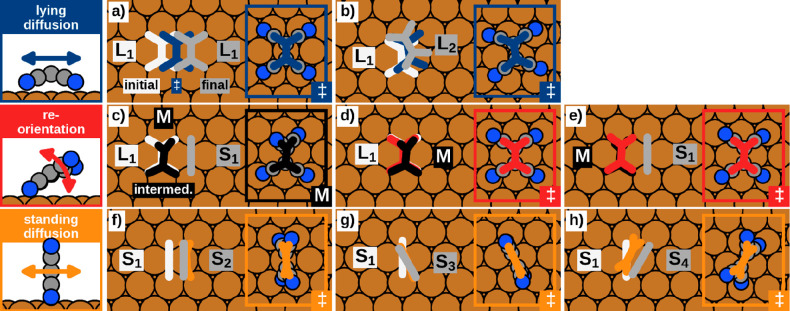
Overview of the elementary transition processes of the three different motion regimes lying diffusion (a,b), reorientation (c–e) and standing diffusion (f–h). In addition to the initial (white) and final (gray) adsorption geometries the positions of the transition states including their specific geometries are provided as well, except for **L**_**1**_ → **S**_**1**_ (c), where instead the obtained intermediate minimum **M** (black) is shown. The relative positions during the transitions itself are presented in a reduced scheme by omitting the nitrogen atoms.

**Figure 4:**
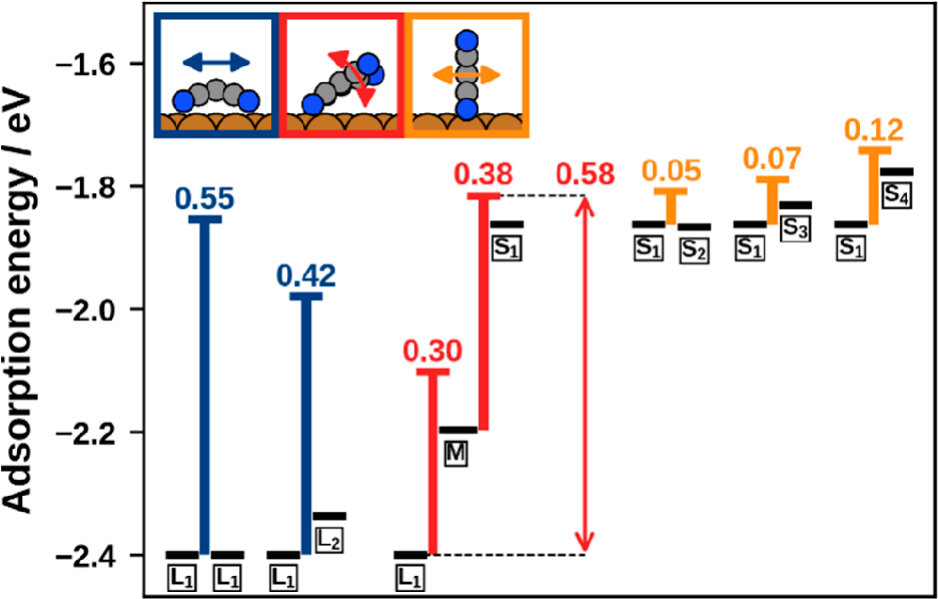
Adsorption energies of initial geometries, transition states, and final geometries of the various transitions. The black bars represent the energies of the local minima, whereas the colored bars represent the energies of the transition states. In addition, barrier heights of the forward transitions are provided. The vertical arrow denotes the effective barrier for reorienting from lying to standing TCNE.

In the course of our computations, we found that 5 of the 11 initialized transitions occur as multistep processes, that is, they include another adsorption geometry and are, therefore, a combination of other transition processes: Two of the three **L**_**1**_ → **L**_**1**_ diffusion transitions (Figure 2b,c) proceed *via* the adsorption geometry **L**_**2**_ and thus reduce to consecutive transitions of **L**_**1**_ → **L**_**2**_ and **L**_**2**_ → **L**_**1**_ (Figure 2d). Therefore, the remaining **L**_**1**_ → **L**_**1**_ transition (Figure 2a) uniquely denotes the direct transition. In addition, the reorientations **L**_**1**_ → **S**_**3**_ (Figure 2f) and **L**_**1**_ → **S**_**4**_ (Figure 2g) proceed *via***S**_**1**_, inferring that the main reorientation process is **L**_**1**_ → **S**_**1**_ (Figure 2e). By investigating **L**_**1**_ → **S**_**1**_ in more detail (discussed later), we also found that this transition proceeds *via* a hitherto overlooked intermediate minimum (**M**). Upright-standing diffusions of **S**_**1**_ to all symmetry equivalent **S**_**1**_ in adjacent adsorption sites (e.g., along the directions ⟨011⟩) occur as multistep processes over **S**_**2**_, **S**_**3**_, and/or **S**_**4**_ (see Supporting Information). All remaining transitions possess exactly one transition state, hence occur as “elementary” transitions. In total, the transition states and minimum energy paths of seven elementary transitions were obtained: Lying TCNE molecules can either diffuse along ⟨011⟩ directions (**L**_**1**_ → **L**_**1**_) or perform rotations (**L**_**1**_ → **L**_**2**_). The observed process of reorientation from a flat-lying to an upright-standing position occurs consecutively *via***L**_**1**_ → **M** and **M** → **S**_**1**_, as discussed later in more detail. For the standing diffusion, the motion in straight lines perpendicular to the molecular plane (similar to a walking motion) is enabled *via***S**_**1**_ → **S**_**2**_, while rotation between the directions [011], [110], and [101̅] of the Cu(111) surface occurs by **S**_**1**_ → **S**_**3**_ and **S**_**1**_ → **S**_**4**_. Figure 3 shows an overview containing the main geometric characteristics. This includes the initial and final adsorption geometries, as well as the positions and the explicit geometries of the obtained transition states. The only exception is the transition **L**_**1**_ → **S**_**1**_ (Figure 3c), where the intermediate minimum **M** is provided instead. The corresponding energy barriers, as well as the absolute adsorption energies of the initial states, the transition states, and the final states are summarized in Table 1 and visualized in Figure 4. The detailed energy paths of all transitions are visualized in the Supporting Information.

**Table 1:** Energetics of the Elementary Transitions^a^

transition	*E*^ini^/eV	*E*^fin^/eV	*E*^‡^/eV	Δ*E*_1_^‡^/eV	Δ*E*_–1_^‡^/eV
**L1→L1**	–2.40	–2.40	–1.85	0.55	0.55
**L1→L2**	–2.40	–2.34	–1.98	0.42	0.36
**L1→M**	–2.40	–2.20	–2.10	0.30	0.10
**M→S1**	–2.20	–1.86	–1.81	0.38	0.05
**S1→S2**	–1.86	–1.87	–1.81	0.05	0.06
**S1→S3**	–1.86	–1.83	–1.79	0.07	0.04
**S1→S4**	–1.86	–1.78	–1.74	0.12	0.03

a Adsorption energies of the initial (*E*^ini^), transition (*E*^‡^) and final state (*E*^fin^) and the corresponding barriers of the forward (Δ*E*_1_^‡^) and the reverse (Δ*E*_–1_^‡^) transition.

**Figure 5:**
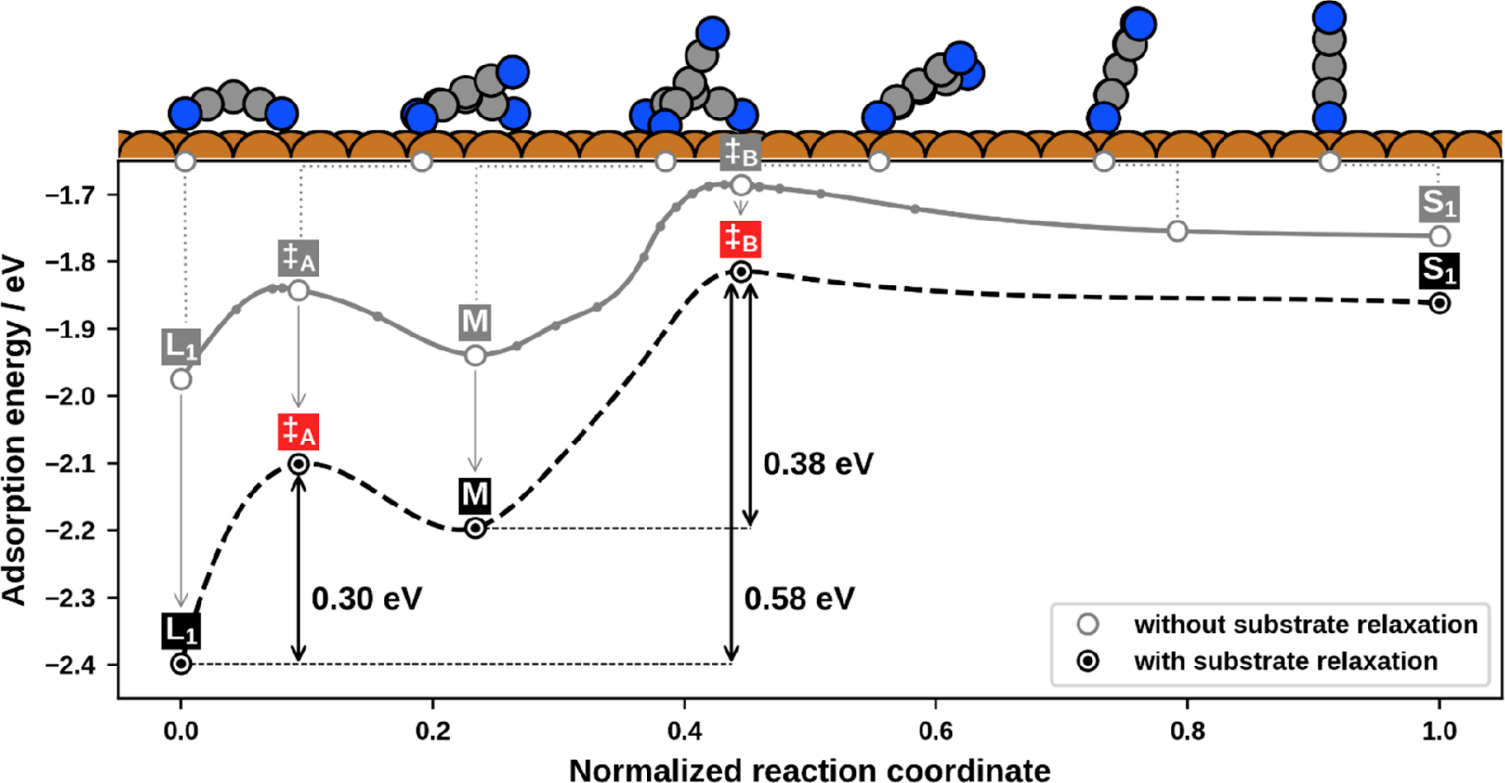
Energy evolution while reorientating from a flat-lying to an upright-standing position with (gray line) and without (black line) constraining the substrate atoms throughout optimizations. The course with the fixed substrate was sampled with 25 images, whereas for the more accurate description, that includes the influence of the substrate, only the minima and the transition states were reoptimized. The interconnecting, dashed line is an interpolation between these reoptimized points. In addition, geometries of characteristic positions are provided in the side view for the initial (**L**_**1**_), intermediate (**M**), and final (**S**_**1**_) minima, as well as the two transition states ‡_**A**_ and ‡_**B**_.

Since the **L**_**1**_ → **S**_**1**_ reorientation process will strongly determine the phase transition versus growth behavior, we discuss this process in more detail. In Figure 5, the energetic and geometric course of the reorientation is visualized. In addition to the path where substrate atoms were included in optimizations (black), also the path obtained by constraining the substrate during optimizations (gray) is shown. Therein, the different shifts of adsorption energies and the change of the energy barrier by 0.2 eV underline that the influence of the substrate is not negligible. In general, the reorientation occurs in a two-step process: The intermediate minimum **M** is 0.20 eV energetically less beneficial than **L**_**1**_. The barrier of **L**_**1**_ → **M** is 0.30 eV, and thus smaller than the barrier of **M** → **S**_**1**_, which amounts to 0.38 eV. The effective total barrier for standing up, that is, the difference between the lowest (**L**_**1**_) and highest point (transition state ‡_**B**_ in Figure 5) of the pathway, is 0.58 eV. For the reverse transition the molecule needs to overcome only a minute barrier of 0.05 eV between **S**_**1**_ and **M**. The backward reaction is completed by overcoming the barrier between **M** and **L**_**1**_ (0.10 eV).

In geometric terms, the reorientation proceeds as follows: The lying TCNE detaches one CN-group, hereafter referred to as “arm”, from the surface before reaching the first transition state (‡_**A**_). The molecule gains stability again at the intermediate minimum (**M**) by repositioning its opposite arm from the top to the hollow site. After the arm next to the already detached one breaks the second CN–Cu bond, both detached arms come closer to each other, until arriving at the second transition state (‡_**B**_). Here the now nearly flat molecule encloses an angle of approximately 30° with the substrate surface. By rotating further into an upright position, the adsorption geometry **S**_**1**_ is reached.

It is likely that the reorientation process of **L**_**2**_ → **S**_**4**_ follows a similar pathway. However, this transition cannot be rate-limiting for the targeted kinetic trapping, because the difference of the adsorption energies of **L**_**2**_ and **S**_**4**_ (0.56 eV) is already as large as the barrier of **L**_**1**_ → **S**_**1**_ (0.58 eV).

### 2.2 Transition Rates

To determine under which conditions the reorientation of TCNE molecules can be prevented, while still allowing for growth of the flat-lying structures, we need to obtain temperature-dependent transition rates by utilizing the energy barriers. We assume that in a hypothetical physical vapor deposition (PVD) experiment an ordered flat-lying structure can form as long as the temperature is sufficiently high for the molecules to readily diffuse. The speed at which this structure grows is then limited by the available material. In a PVD experiment, this is given by the rate at which TCNE molecules are deposited onto the substrate. Furthermore, it is plausible to assume that the structure becomes (kinetically) stabilized once it reaches mesoscopic dimensions or becomes buried under a significant amount of material, that is, once the deposited TCNE is several layers thick. In other words, if the growth to multilayers occurs faster than the time required for even a single TCNE to reorient into an upright position, we assume to have kinetically trapped the flat-lying structure. In short, we need to find a temperature range where (a) the diffusion of molecules is much faster and (b) the reorientation is considerably slower than a given deposition rate.

We can calculate temperature-dependent transition rates *k*(*T*) utilizing the harmonic transition state theory (see Methods for details) with energy barriers Δ*E*^‡^ from Table 1 and attempt frequencies *A*, as provided in Table 2:
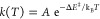
1Since our goal is to prevent the reorientation of individual molecules to the upright-standing position, we want to discuss a joint process of the reorientation (**L**_**1**_ → **S**_**1**_) rather than the separate elementary transitions **L**_**1**_ → **M** and **M** → **S**_**1**_. Thus, we assign an effective barrier of 0.58 eV (see Figure 5) to the joint process of standing up. For the lying down, **S**_**1**_ → **M** is the decisive step (barrier of 0.05 eV).

**Table 2:** Attempt Frequencies Obtained by Means of Harmonic Transition State Theory^a^

	**L**_**1**_ → **L**_**1**_	**L**_**1**_ → **L**_**2**_	**L**_**1**_ → **S**_**1**_	**S**_**1**_ → **S**_**2**_	**S**_**1**_ → **S**_**3**_	**S**_**1**_ → **S**_**4**_
*A*_1_/Hz	2.0 × 10^15^	2.9 × 10^14^	5.0 × 10^13^	1.7 × 10^12^	1.0 × 10^12^	1.7 × 10^13^
*A*_–1_/Hz	2.0 × 10^15^	1.3 × 10^14^	8.8 × 10^11^	4.6 × 10^12^	1.2 × 10^12^	1.3 × 10^13^

a *A*_1_ and *A*_–1_ denote attempt frequencies of forward and reverse transitions, respectively.

Apart from energy barriers, rates are determined by the attempt frequencies of the transitions. In principle, attempt frequencies are the vibration frequencies in direction of the reaction coordinate. Within harmonic transition state theory, they are explicitly obtained from the stable vibration frequencies of the initial and the transition states (for details see Methods). As Table 2 shows, the attempt frequencies are very different for different processes, covering 5 orders of magnitude. The joint process of lying down (**S**_**1**_ → **L**_**1**_) has with 8.8 × 10^11^ Hz the lowest attempt frequency, while the largest is obtained for the **L**_**1**_ → **L**_**1**_ diffusion with 2.0 × 10^15^ Hz. In total, attempt frequencies for the lying diffusion are by up to 3 orders of magnitude larger than the ones of the standing diffusion. For the reorientation process, attempt frequencies of the standing up are about 100 times larger than the attempt frequencies of falling over.

**Figure 6:**
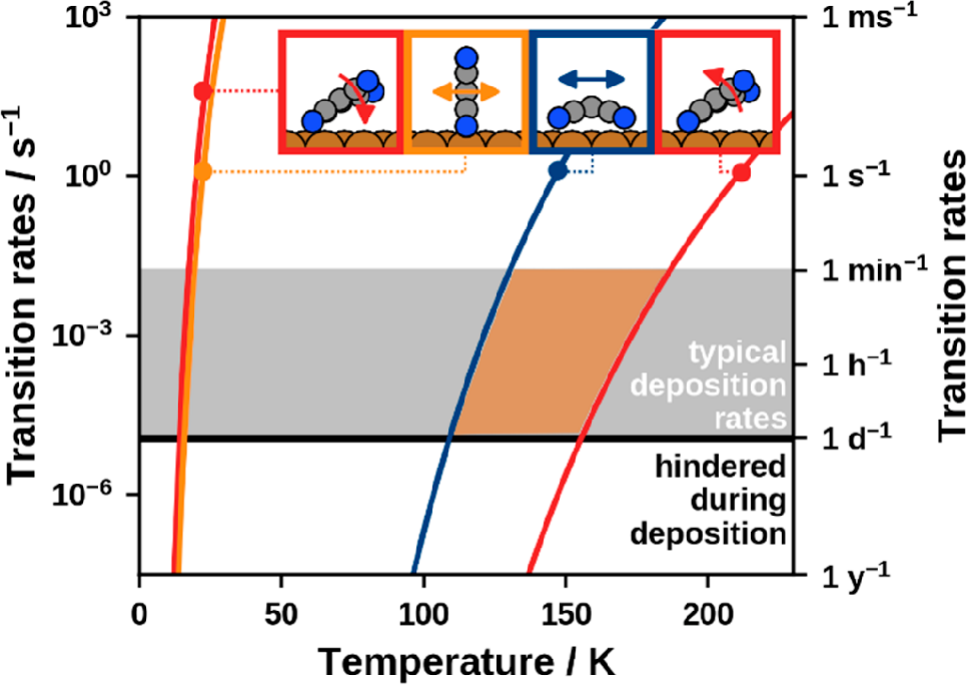
Limiting rates of lying diffusion **L**_**1**_ → **L**_**2**_ (blue), reorientation **L**_**1**_ ↔ **S**_**1**_ (red) and standing diffusion **S**_**1**_ → **S**_**2**_ (orange) as a function of the process temperature. The range of typical deposition rates is marked by the gray area, and the limiting transition rate for hindering distinct processes during deposition is indicated by the black line at 1 transition per day. Within the former, the area where the monolayer of lying molecules is kinetically trapped against reorientation is highlighted.

Using these attempt frequencies, the temperature-dependent transition rates are calculated according to eq 1 and shown in Figure 6. Before explicitly investigating the rates of the single processes, we discuss at which rates we can consider transitions to be suppressed within the growth process. Within PVD experiments, thin films (i.e., multilayers) of organic materials are typically deposited within minutes to (at most) days. This corresponds to deposition rates *k*_dep_ on the order of 1 monolayer per minute to 1 monolayer per day. On the basis of the obtained transition rates (Figure 6), we can identify at which temperatures individual processes occur much more slowly than the deposition process itself. In other words: We can consider single processes as suppressed if transition rates *k* < *k*_dep_ are enforced. For the present discussion, we propose a target transition rate *k* of 1 transition per day (10^–5^ transitions per second), as indicated by the black line. The temperatures required to reach this target transition rate are stated in Table 3. For a concise representation, only the limiting transitions, that is, the transitions with the highest rates within a class, of the lying diffusion (**L**_**1**_ → **L**_**2**_), the standing diffusion (**S**_**1**_ → **S**_**2**_), the standing-up (**L**_**1**_ → **S**_**1**_) and the lying-down (**S**_**1**_ → **L**_**1**_) are plotted. In the Supporting Information a visualization of the rates of all transitions is provided, as well as a detailed uncertainty discussion including root-mean-square uncertainty estimates of the obtained suppression temperatures. Summarizing the outcome, we estimate the uncertainty of the suppression temperatures of the lying diffusion to ≈30 K, whereas the one for standing up is with ≈40 K the largest error estimate. For all other transitions the uncertainty is ≈20 K.

**Table 3:** Estimated Temperatures for Suppression^a^

	**L**_**1**_ → **L**_**1**_	**L**_**1**_ → **L**_**2**_	**L**_**1**_ → **S**_**1**_	**S**_**1**_ → **S**_**2**_	**S**_**1**_ → **S**_**3**_	**S**_**1**_ → **S**_**4**_
*T*_1_/K	140	110	160	20	20	30
*T*_–1_/K	140	95	10	20	10	10

a *T*_1_ and *T*_–1_ refer to the temperatures of suppression of forward and reverse transitions, respectively. Hereby, transitions are considered being suppressed for a rate of 1 transition per day.

As shown in Figure 6, the diffusion of standing TCNE along single symmetry axes (**S**_**1**_ → **S**_**2**_) and the lying-down (**S**_**1**_ → **L**_**1**_) are the fastest processes and exhibit similar rates. Therefore, standing molecules might not diffuse over long distances before falling over again. For temperatures above 100 K all processes of standing diffusion and the lying-down occur on subnanosecond time scales. The diffusion of the flat-lying molecules freezes out at temperatures below 110 K. This temperature is relatively high due to concurrent relaxations of the substrate, specifically a “pulling out” of Cu atoms bonded to the nitrogen atoms by 0.2 Å. This relaxation increases the barrier by 0.19 eV. Without it, the diffusion would freeze out at temperatures below 60 K. For temperatures above 140 K, the lying diffusion proceeds on the order of seconds, which increases to the order of microseconds at room temperature. Finally, the process of standing up (**L**_**1**_ → **S**_**1**_) is the slowest. For temperatures below 160 K we estimate a rate of less than one transition per day. At room temperature it still occurs very efficiently (millisecond time scale). Here, we remind the reader that this joint process is, indeed, a two-step process. For the sake of completeness, the rates for these two elementary processes are provided in the Supporting Information (Figure S7).

On the basis of these results, we predict that, in the temperature range of 110 to 160 K, the standing up of individual molecules can be suppressed while molecules can still diffuse.

Suggesting a process temperature of 140 K, lying molecules diffuse by a rate of ≈0.2 transitions per second. This should be sufficiently high to ensure a diffusion mobility that allows building at least a full monolayer of lying TCNE within the deposition time. For lower temperatures, growth of ordered structures is likely to be inhibited by random aggregation of impinging molecules. For 140 K, the rate of standing up is 0.3 transitions per year. Once they are standing, the molecules fall down again within nanoseconds. Therefore, it is unlikely that standing seeds are created during the growth process, provided that deposition rates are low enough to avoid aggregation. For this regime, detailed knowledge of the influence of standing seeds on the stability of standing molecules becomes dispensable.

At first sight, this prediction is qualitatively not consistent with the experiments of *Erley and Ibach*, who observed both, standing and lying molecules for deposition at 100 K.^20,37^ While the formation of lying seeds is covered within the estimated uncertainty, standing seeds should not form according to our predictions. This contradiction might result from (a) an unrecognized temperature increase in the experiment of *Erley and Ibach* during the highly exothermal deposition process and/or (b) from the reduction of the energy barrier caused by collaborative effects.

For coverages that exceed the one of the favored flat-lying structure (i.e., when a second layer is created) we assume in the first approximation of non-interacting adsorbates that the reorientation of the whole monolayer can be suppressed as well for temperatures below 160 K. Despite reorientation rates will increase when taking these interactions into account, we expect to prevent the reorientation of the whole first layer by depositing further layers fast enough until the layer thickness reaches a mesoscopic scale. To check whether this assumption holds true detailed investigations about the intermolecular and interlayer processes will need to be performed in the future.

## 3 Conclusion

To propose experimental conditions that prevent the reorientation of flat-lying molecules in the first adsorbate layer to the thermodynamically favored upright-standing positions, we studied kinetic processes of tetracyanoethylene (TCNE) molecules on a Cu(111) surface. Utilizing the nudged elastic band method and the harmonic transition state theory, energy barriers and transition rates were obtained for the diffusion of lying and standing TCNE molecules, as well as for the reorientation between these two positions. The most dominant and thus limiting reorientation process turned out to advance in two steps, exhibiting an effective energy barrier of 0.58 eV for standing up and 0.05 eV for lying down. On the basis of the obtained rates, we estimate that for temperatures above 110 K a sufficiently high diffusion mobility is ensured, which further allows the formation of an ordered monolayer of flat-lying TCNE. While our investigation reveals that individual molecules can be prevented from standing up for temperatures below 160 K, this finding offers an initial indication for the reorientation behavior of the whole monolayer. Determining this temperature more precisely and assessing how long the first layer remains kinetically trapped upon deposition of further layers will require further studies on the intermolecular and interlayer processes. Nevertheless, this work constitutes a first step toward fully understanding transition processes of organic thin films.

## 4 Methods

In this work, the sampling of the potential energy surface is conducted within the framework of Kohn–Sham density functional theory as implemented in the software package FHI-aims.^38^ We use the PBE^39^ functional and the TS^surf^ dispersion correction.^40^ We apply the repeated slab approach with periodic boundary conditions in all three dimensions. Unit cell heights of 68 Å ensure vacuum heights of at least 50 Å between two consecutive slabs. Hereby a dipole correction^41^ is used to electrostatically decouple the replicas in the *z*-direction. The TCNE molecules are placed on a substrate consisting of seven copper layers with a lattice constant of 2.55 Å (which was obtained by a Birch–Murnaghan fit). The band structure is sampled using a generalized Monkhorst–Pack grid^42−44^ with a spacing of  nm^–1^. A Gaussian broadening of 0.1 eV is applied to all states.

FHI-aims employs numeric atom-centered basis functions. In this work, we use the “tight” default settings for C and N. The three uppermost Cu layers are also treated with the “tight” species defaults, whereas the residual four layers are treated with “light” settings to save computational time. This is described in detail in the Supporting Information of a previous publication,^20^ in which identical DFT settings are used.

The convergence criteria of the SCF-procedure were set to 1 × 10^–2^ eÅ^–3^ for the charge density, 1 × 10^–5^ eV for the energy and 1 × 10^–3^ eV Å^–1^ for the forces.

To achieve converged adsorption energies (within ≈30 meV), 6 × 6 Cu supercells are required.

The resulting energies correspond to electronic energies of the whole system *E*_sys_ at zero Kelvin. Adsorption energies *E*_ads_ are determined according to eq 1, where *E*_mol_ is the energy of a relaxed molecule in the gas phase and *E*_sub_ is the energy of the prerelaxed substrate, as used in the slab.
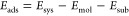
2By this definition, more favored adsorption geometries are connected to more negative energies.

While molecular dynamics simulations are the standard method for kinetic studies, their application is not affordable for the investigated system and purpose. As timesteps for similar systems are typically in the order of femtoseconds or less, the available simulation time is not sufficient for reliably escaping basins of a wide area of attraction to measure barriers and rates of processes such as reorientations. The computational cost is further increased by sampling the potential energy surface on the level of density functional theory, which is necessary to capture the underlying chemistry. Even though advances in accelerating sampling of rare events^45−47^ have been made, we decided on using transition path sampling methods instead. Transition rates between single adsorption minima are provided *via* harmonic transition state theory, whereas energy barriers themself are determined beforehand with a transition state search method.

For the transition state search, the climbing image nudged elastic band (CI-NEB) method^21,22^ augmented with the fast inertial relaxation engine (FIRE) optimizer^48^ is applied. The workflow can be summed up as the following: The transition path is initialized in a 4 × 4 supercell between the selected pair of minima with up to five images using the image dependent pair potential (IDPP) method^49^ as shipped by the software package ASE.^50^ After several iterations, only the images with the highest energies and/or forces are updated for efficiency reasons. Once the NEB force of the image with the highest energy drops below 0.01 eV/Å, further images are inserted and converged to verify that the highest barrier along the path is found. All transition paths are sampled by 7 to 25 images with residual NEB force <0.05 eV/Å, whereas the NEB forces of all transition states are ≤0.01 eV/Å. In addition, all transition states are reoptimized in a 6 × 6 supercell where the atoms of the two uppermost copper layers are set unconstrained: At first only the substrate is allowed to relax, while afterward the transition state is reoptimized by unconstraining both the adsorbate and the substrate to NEB forces < 0.01 eV/Å. The transition state is, per definition, a first order saddle point, where the Hessian exhibits exactly one instable eigenmode that corresponds to a negative curvature or eigenfrequency.

Numerical vibrational analyses were performed (Γ-point, 4 × 4 supercell, fixed substrate) for all minima and transition states, in order to (a) ensure that the transition states have only one negative frequency, and (b) to obtain the attempt frequencies required for the harmonic transition state theory. Displacements of 0.01 Å are applied for computing Hessians. In the Supporting Information, we explain why Hessians are symmetrized and how additional instable frequencies at transition states and minima are treated.

The harmonic transition state theory^25,26^ enables determining the transition rates *k* as stated in eq 4.
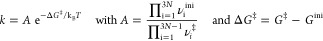
4Therein, *k* is the product of the harmonic attempt frequency *A* and the Boltzmann-factor containing the Gibbs free energy barrier Δ*G*^‡^ and the temperature *T*. Δ*G*^‡^ is the difference of the Gibbs free energy of the transition state (*G*^‡^) and the initial state (*G*^ini^), and in general also depends on the electronic energy, the temperature, the pressure, and the unit cell size. In this study, however, the unit cell size and the number of adsorbate molecules per unit cell stay unchanged for all transitions which reduces the dependency of Δ*G*^‡^ to the pure electronic energy barrier Δ*E*^‡^ (see Supporting Information for details). *A* is the ratio of the products of the stable vibration frequencies ν_i_ at the initial state and the transition state.

## Supporting Information Available

Thermodynamically stable monolayers, energy courses of elementary transitions, transition rates of all transitions (elementary and joint processes), and detailed uncertainty discussion of the temperatures required to suppress distinct processes (PDF)

## Notes

*Data Availability*: The calculations used in this manuscript are available in the NOMAD database (10.17172/NOMAD/2021.10.07-1).
