# Supplementary material for: Toward Targeted Kinetic Trapping of Organic–Inorganic Interfaces: A Computational Case Study
Source: ACS Phys Chem Au. 2021 Oct 11;2(1):38–46. doi: 10.1021/acsphyschemau.1c00015 (PMC8796281; doi:10.1021/acsphyschemau.1c00015)
Supplement: Supplementary file 1 — pg1c00015_si_001.pdf [file pg1c00015_si_001.pdf]

Supporting Information

for

"Toward Targeted Kinetic Trapping of Organic-Inorganic Interfaces:

A Computational Case Study"

Anna Werkovits, Andreas Jeindl, Lukas Hörmann, Johannes J. Cartus, Oliver T. Hofmann*
Institute of Solid State Physics, TU Graz, NAWI Graz, Petersgasse 16/II, 8010 Graz, Austria

Table of Content

1 Numerical convergence of DFT calculations 2
2 Thermodynamically stable monolayers of TCNE/Cu(111) 2
3 Transitions 3
3.1 Example for identification of non-elementary transition: $S_1 \rightarrow S_1$ transition 3
3.2 Elementary transitions 4
4 Transition rates 5
4.1 Treatment of the Gibbs free energy 5
4.2 All transition rates 5
4.2 Joint process of reorientation 6
5 Uncertainty discussion 7
5.1 Transition states and energy barriers 7
5.2 Vibration frequencies 9
5.2.1 Asymmetricity 9
5.2.2 Quality of transition states and minima 9
5.2.3 Uncertainty estimation of vibrational frequencies 12
5.3 Attempt frequencies 13
5.4 Suppression temperatures 13
6 References 15

1 Numerical convergence of DFT calculations

In order to investigate individual molecules by utilizing the repeated slab approach, convergence tests of the super cell size are conducted. In detail, the convergence of the adsorption energies of three representative geometries on a fixed substrate (flat-lying: **L_1**, upright-standing: **S_1** and their respective transition state) and the corresponding energy barrier (**$L_1 \rightarrow S_1$**) are visualized in Figure S1. Based on these results and a trade-off between numerical accuracy and computational effort, final results are obtained in a 6x6 super cell. Accordingly, numerical accuracies of adsorption energies amount ≤ 0.03 eV, whereas the energy barrier of **$L_1 \rightarrow S_1$** is considered to be converged within 0.01 eV.

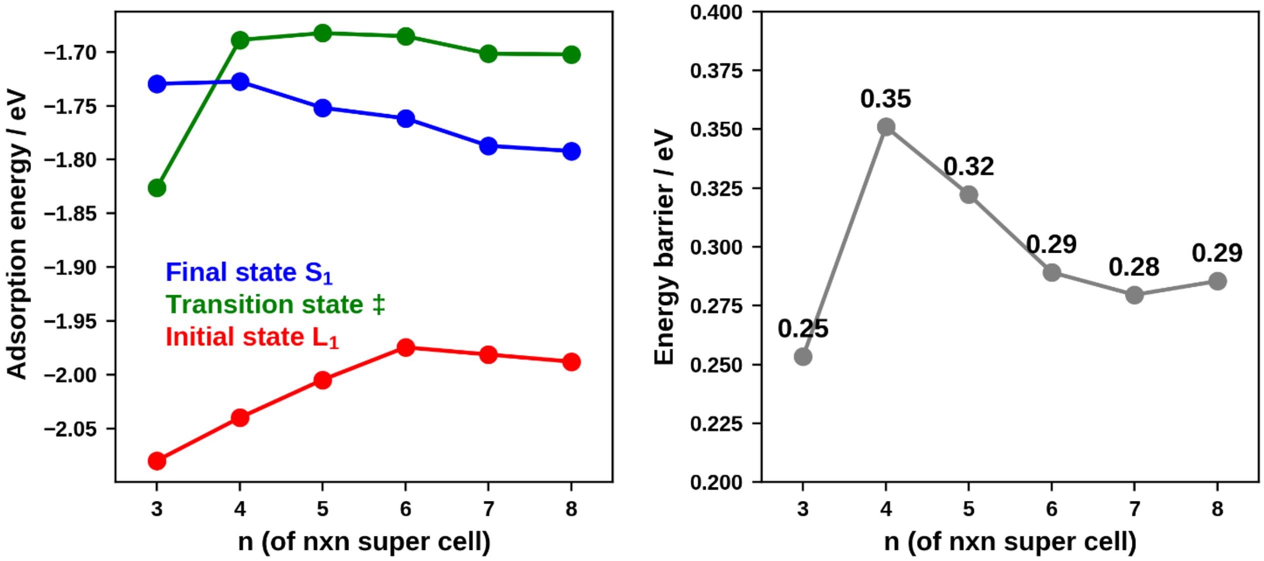

Figure S1: *Numerical convergence of adsorption energies (left) and energy barriers (right) with respect to the super cell size.*

2 Thermodynamically stable monolayers of TCNE/Cu(111)

As predicted by Egger et al.,[1] there are two favorable structures for different coverage ranges: For low coverages, the flat-lying structure from Figure S1 is thermodynamically the most favorable, whereas for increasing coverages the herringbone structure with upright-standing molecules (Figure S2) becomes superior.

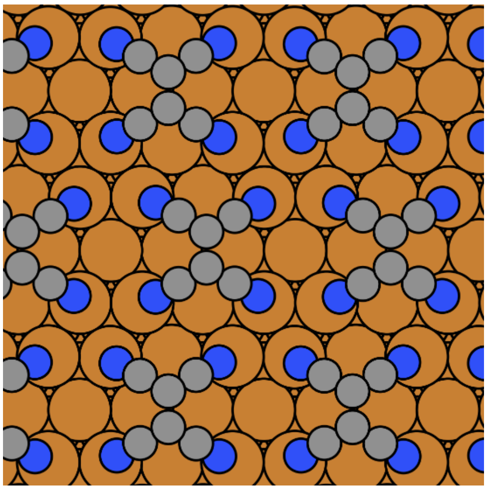

Figure S2: *Flat-lying monolayer*

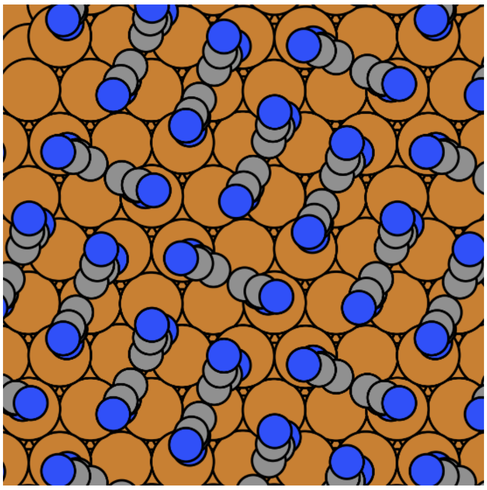

Figure S3: *Upright-standing monolayer*

3 Transitions

3.1 Example for identification of non-elementary transition: $S_1 \rightarrow S_1$ transition

In Figure S3 the transition $S_1 \rightarrow S_1$ is initialized in linear fashion. After some iterations of the nudged elastic band method, we see in Figures S4 and S5 that the central image converged towards S2. Therefore, splits in the equivalent transitions $S_1 \rightarrow S_2$ and $S_2 \rightarrow S_1$.

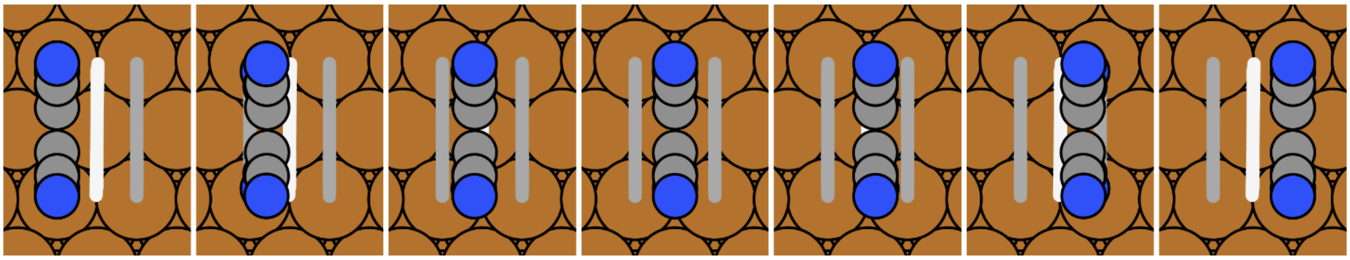

Figure S3: *Initialized $S_1 \rightarrow S_1$ transition*

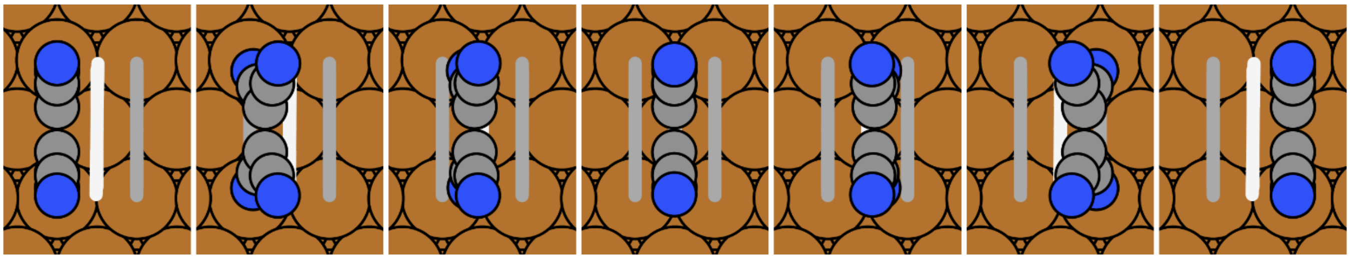

Figure S4: *$S_1 \rightarrow S_1$ transition after 74 iterations of the nudged elastic band method*

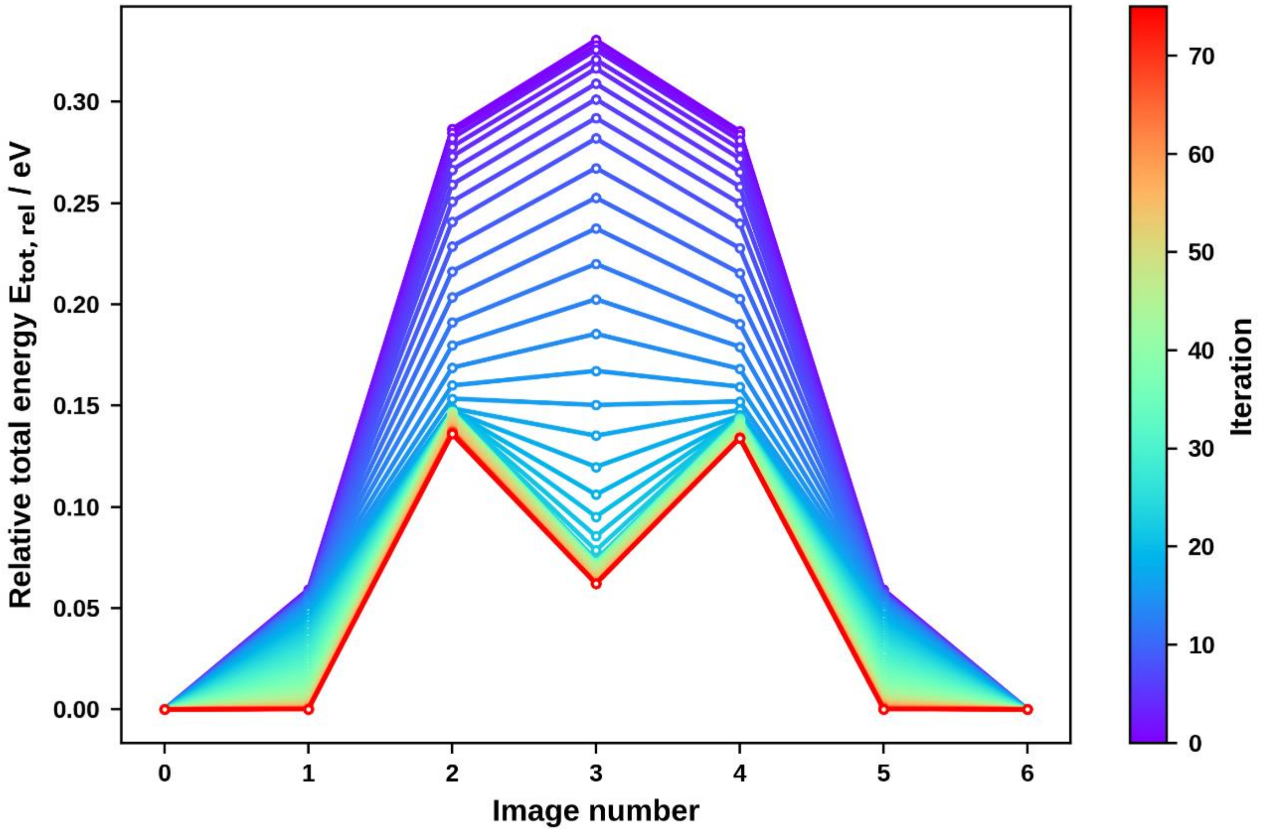

Figure S5: *Energy evolution of the $S_1 \rightarrow S_1$ transition after applying the nudged elastic band method*

3.2 Elementary transitions

Figure S6 depicts changes in adsorption energy in dependence of the normalized reaction coordinate for all transitions. In addition, the influence of including substrate relaxations to the optimizations is visualized.

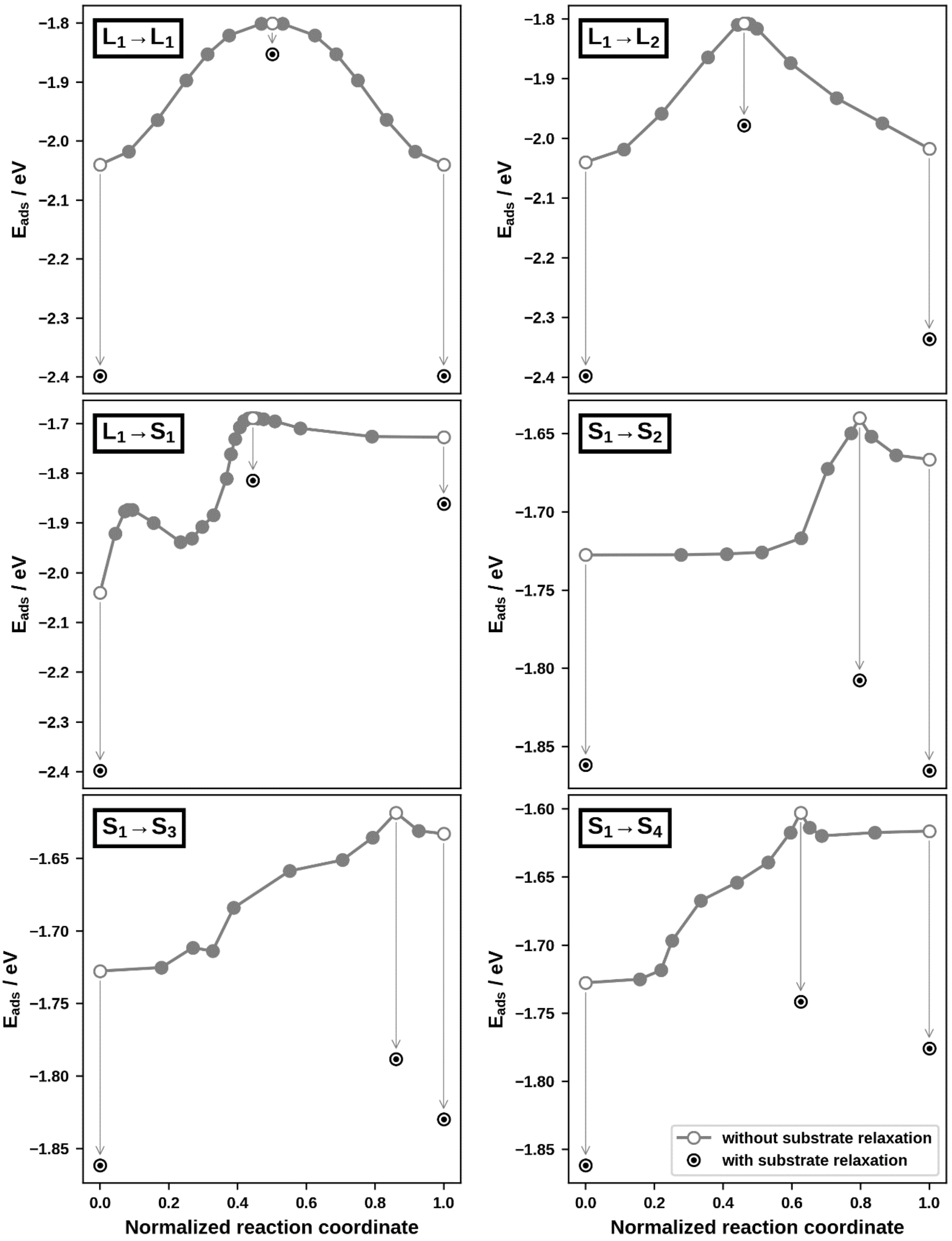

Figure S6: *Sampled minimum energy paths described by the change of adsorption energy along the normalized reaction coordinate. The paths in gray are simulated in a 4x4 super cell and a fixed substrate, whereas the black dots show the change after re-optimization in a 6x6 super cell by including substrate relaxation.*

4 Transition rates

4.1 Treatment of the Gibbs free energy

We apply *ab initio* thermodynamics[2] to include finite temperature effects to the energies as obtained via density functional theory. For modelling growth experiments as closed systems at constant temperature and pressure, the Gibbs free energy is the relevant thermodynamic potential. In Equation 1, the Gibbs free energy of adsorption is constructed from the adsorption energy E_{ads} and the contribution of the chemical potential μ_{ads} , while mechanical work, configuration entropy and vibration enthalpy are neglected, as it is commonly done in literature.[2]

$$G_{\mathrm{ads}} = E_{\mathrm{ads}} - \mu_{\mathrm{ads}} N_{\mathrm{ads}} \qquad \text{(Equation 1)}$$

N_{ads} is the number of molecules adsorbed per unit cell, whereas μ_{ads} is the chemical potential of the molecule in the gas phase, that depends on temperature and pressure. μ_{ads} is obtained in the ideal gas approximation by simply using translational and rotational contributions as provided in the thermochemistry package distributed within ASE[3].

For our case, all unit cells are identical and include only one adsorbate, i.e. $N_{ads} = 1$. Therefore, μ_{ads} is a global constant and can be neglected and G_{ads} directly reduces to E_{ads}.

4.2 All transition rates

As only the joint processes and not the elementary transitions of the reorientation are relevant for predicting process conditions for kinetically trapping the first layer of flat-lying molecules, the attempt frequencies of the two elementary steps of the reorientation process are not stated in the main manuscript. Thus, these are provided in Table S1.

Table S1: *Attempt frequencies obtained by means of harmonic transition state theory. The subscripts 1 and -1 denote forward and reverse transitions, respectively.*

	**$L_1 \rightarrow M$**	**$M \rightarrow S_1$**
**A_1 / Hz**	1.7×10^{13}	5.0×10^{13}
**A_{-1} / Hz**	9.0×10^{12}	8.8×10^{11}

In Figure S7, also the transition rates of all elementary transitions and joint processes are visualized. Figure S8 shows the same content, but in a wider range.

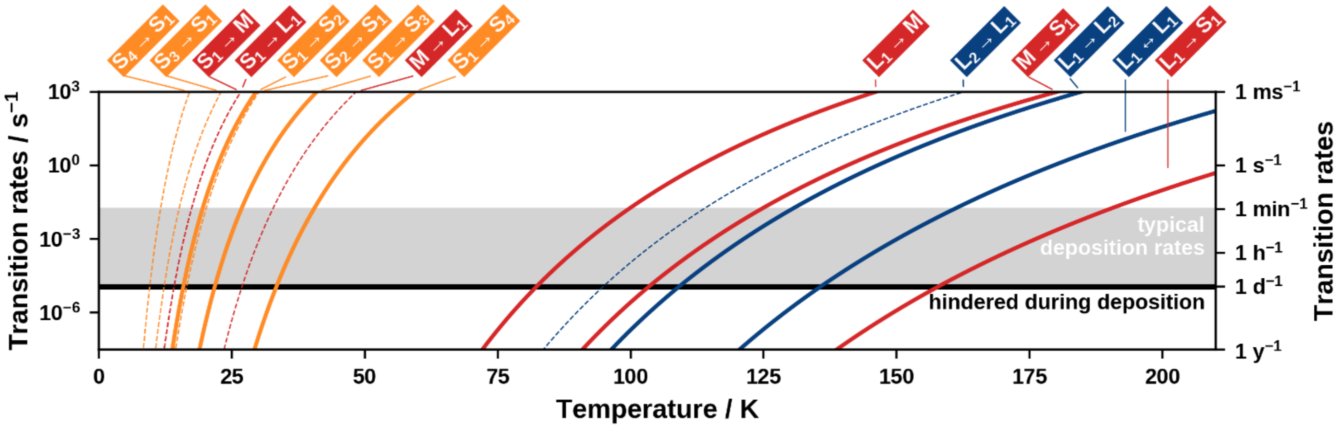

Figure S7: *Transition rates in dependence of temperature including the two elementary transitions of the reorientation*

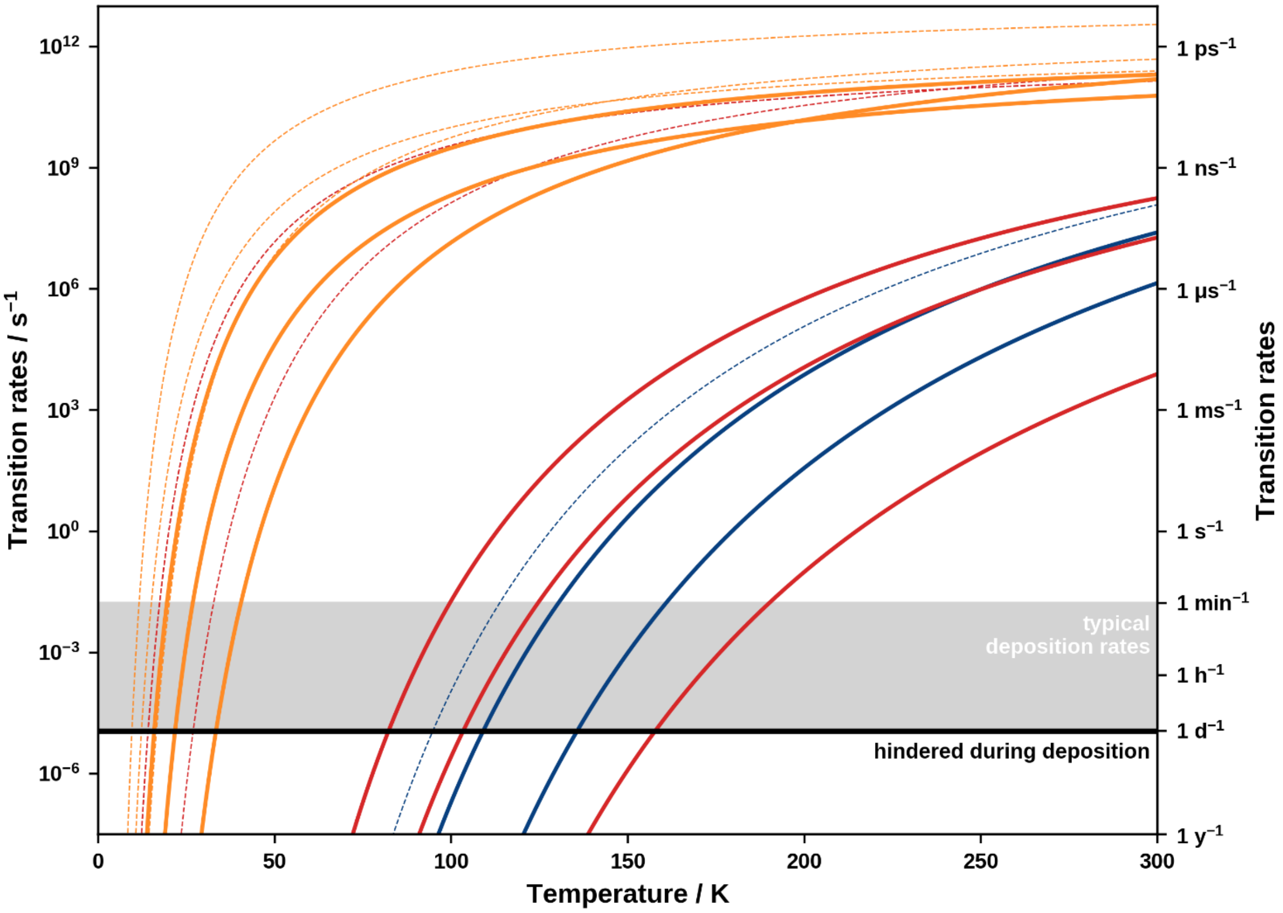

Figure S8: *Transition rates in dependence of temperatures in an extended range (labeling according to Figure S7)*

4.2 Joint process of reorientation

Since our goal is to prevent the reorientation of individual molecules to the upright-standing position, we want to discuss a joint process of the reorientation rather than the separate elementary processes.

For the joint process of standing-up ("$L_1 \rightarrow S_1$") the rate determining transition state is the one of step $M \rightarrow S_1$. This is evident in Figure S7. Thus, we assign an effective barrier of 0.58 eV to the joint process of standing-up, as indicated in Figure 5 of the main manuscript. This is constituted of the sum of the pure difference in adsorption

energies of $L_1 \rightarrow M$ (0.20 eV) and the energy barrier of $M \rightarrow S_1$ (0.38 eV). This description is formally valid under the assumption that L_1 and M are in a pre-equilibrium. In addition, the attempt frequency of $M \rightarrow S_1$ (5.0×10^{13} s^{-1}) is used.

For the joint process of lying down ("$S_1 \rightarrow L_1$"), we are especially interested in the step $S_1 \rightarrow M$. Therefore, we employ the $M \rightarrow L_1$ barrier of 0.05 eV and its attempt frequency (8.8×10^{11} s^{-1}).

5 Uncertainty discussion

In general, there are several sources of uncertainty or errors arising from the applied DFT functional, geometric constraints, the transition state search, the transition state theory and the thermodynamic description.

Here, we discuss how these uncertainties influence the uncertainty of the temperatures that are required for suppression of single elementary or joint processes. In detail, we include the uncertainty of the transition states and especially the influence from the vibrational frequencies.

5.1 Transition states and energy barriers

The quality of the obtained transition states is mainly influenced by the accuracy of the DFT calculation (≤ 30 meV, see Section 1), as well as by the performance of the nudged elastic band method (NEB) method. In general, the geometric path used for the initialization of the NEB method can influence the resulting minimum energy path. Therefore, the path converges either to the global or only to a local solution. Checking the stability of the resulting transition paths for different initial paths is not affordable. Besides this influencing factor, the sampling resolution along the obtained path is important as well: To ensure that the highest barrier of the resulting path is captured, a plausible resolution has to be achieved. In addition, the transition state should be sampled as accurately as possible for a trustworthy measure of the energy barrier and the vibrational frequencies required for computing transition rates. Trading off the accuracy with the involved cost, we tried to push the residual NEB forces F_{NEB} on the transition state below 0.01 eV Å^{-1}. The resolution of the chain of images is expressed via the maximal distance Δx atoms of the adsorbate exhibit between two neighbored images. For a resolution of $\Delta x = 1$ Å, this would refer to a maximal uncertainty of the transition state energy of $\Delta E = F_{\mathrm{NEB}} \cdot \Delta x = 10$ meV.

In Table S2, the number of images per run, the maximal residual NEB forces of the whole chain and especially for the obtained transition state are provided, as well as the resolution at the transition state. Residual forces at the transition states of < 0.01 eV Å^{-1} could be obtained. The resolution at the transition state is around 0.05 to 1.00 Å. When accounting for the whole chain of images for all calculated transitions, the resolution ranges from 0.04 to 1.92 Å. Here, we remind the reader that these calculations have been conducted in a 4x4 super cell.

Table S2: *Force accuracies and information about resolution of the conducted NEB runs in a 4x4 super cell. The number of sampled images is n_{images}. The absolute value of the maximal NEB force acting on a single atom of TCNE along the sampled transition path is denoted as $|F|_{NEB,max}$, whereas $|F|_{NEB,max,TS}$ states the maximum force acting on the transition state. The resolution of the sampled images is expressed via the maximum distance atoms of the adsorbate exhibit between two neighbored images. Here, $\Delta x_{max,pre\ TS}$ and $\Delta x_{max,post\ TS}$ provide the resolution at the transition state.*

	n_{images}	$\|F\|_{NEB,max}$ / eV Å^{-1}	$\|F\|_{NEB,max,TS}$ / eV Å^{-1}	$\Delta x_{max,pre\ TS}$ / Å	$\Delta x_{max,post\ TS}$ / Å
**$L_1 \rightarrow L_1$**	15	1.8E-02	3.5E-03	0.10	0.09
**$L_1 \rightarrow L_2$**	13	3.0E-02	2.0E-03	0.10	0.05
**$L_1 \rightarrow M$**	7	1.4E-02	4.8E-03	0.25	1.00
**$M \rightarrow S_1$**	19	4.6E-02	4.3E-03	0.09	0.10
**$S_1 \rightarrow S_2$**	11	3.9E-02	1.2E-02	0.23	0.22
**$S_1 \rightarrow S_3$**	11	3.0E-02	2.7E-03	0.36	0.30
**$S_1 \rightarrow S_4$**	13	3.9E-02	6.9E-03	0.27	0.29

In Section 5.2.2 Figure S10 we will show that the uncertainty of the transition state energy is rather < 1 meV than < 10 meV for the 4x4 super cell. Nevertheless, the transition states have been re-optimized in a 6x6 super cell in two steps. Firstly, the atoms of the two uppermost copper layers of the transition state were relaxed. Secondly, a single-image NEB run was conducted for an unconstrained motion of both, the substrate and the adsorbate atoms. Except for the $L_1 \rightarrow L_1$ transition, the convergence threshold of 0.01 eV Å^{-1} had already been reached with the first step.

In Section 1, the uncertainty of the **$L_1 \rightarrow S_1$** energy barrier (with a fixed substrate) was quantified to be ≈ 0.01 eV. But as it is not sure that this is valid for all other barriers and including the effects of unconstraining the substrate at optimizations, we assign a higher uncertainty of 0.06 eV to the energy barriers, i.e. the maximum error one obtains by calculating differences of adsorption energies with an uncertainty of 30 meV.

5.2 Vibration frequencies

For the computation of vibrational analyses at the obtained minima and transition states displacements of 0.01 Å are utilized. In addition, two corrections regarding the symmetry of the Hessian and the quality of low and instable frequencies are applied as discussed in Section 5.2.1 and 5.2.2, respectively. Furthermore, relative uncertainties of vibration frequencies are estimated as well in Section 5.2.3. We remind the reader that these calculations have been conducted in a 4x4 super cell.

5.2.1 Asymmetricity

As Hessians are obtained numerically, they are not perfectly symmetric. Therefore, they are symmetrized. To quantify these asymmetries the mean and the maximal value of the absolute differences of the original and the symmetrized Hessian is calculated. Tables S3 and S4 show that the mean of the asymmetricity ranges from 0.02 to 0.03 eV $Å^{-2}$, whereas the maximal values lie between 0.09 and 0.27 eV $Å^{-2}$ for minima and transition states.

Table S3: *Asymmetry of Hessian matrices for the minima.*

**Minimum**	**L_1**	**L_2**	**M**	**S_1**	**S_2**	**S_3**	**S_4**
**RMS / eV $Å^{-2}$**	0.02	0.02	0.03	0.02	0.02	0.02	0.03
**Max / eV $Å^{-2}$**	0.10	0.09	0.27	0.09	0.15	0.14	0.34

Table S4: *Asymmetry of Hessian matrices of the transition states.*

**Transition state**	**$L_1 \rightarrow L_1$**	**$L_1 \rightarrow L_2$**	**$L_1 \rightarrow$ M**	**M $\rightarrow S_1$**	**$L_1 \rightarrow S_1$**	**$S_1 \rightarrow S_2$**	**$S_1 \rightarrow S_3$**	**$S_1 \rightarrow S_4$**
**RMS / eV $Å^{-2}$**	0.02	0.02	0.02	0.03	0.02	0.02	0.02	0.02
**Max / eV $Å^{-2}$**	0.10	0.10	0.10	0.27	0.10	0.09	0.09	0.09

5.2.2 Quality of transition states and minima

After the NEB run converges to the defined convergence criteria, we perform vibrational analyses to ensure that the obtained transition state is indeed a 1st order saddle point. In theory, the vibrational analysis must have exactly one instable (negative) vibration frequency. Similarly, minima must obtain only stable (positive) vibration frequencies. In practice, sometimes small, but positive, frequencies are erroneously identified as additional instable frequencies. This is a common issue inherent to density functional theory originating from numerical integration on a finite k-point grid.[4] In order to check if the unwanted negative frequencies result from this issue or if the sampled transition state (or minimum) is a higher order saddle point, the potential energy surface is distinctly sampled along all instable vibration modes. In detail, displacements (perturbations) along these modes in the range of ± 0.1 Å are applied. The displacements are defined as the maximal displacement of the atoms in this vibration mode.

As schematically visualized in Figure S9, this easily allows then to compare the harmonically approximated vicinity of the potential energy surface along the vibration mode (as probed by the vibration analysis) with the directly calculated energy course.

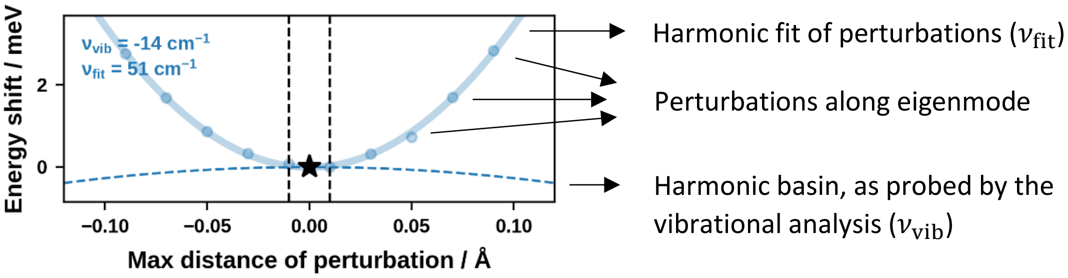

Figure S9: *Example for an erroneously obtained instable vibration frequency.*

By checking the consistency of the resulting energy course (minimum, maximum etc.) it becomes clear whether these negative modes occurred from the mentioned numerical inaccuracy or if the instable frequency correctly represents an extremum with a negative curvature. In case of the latter, the transition state search or geometry optimization must be continued and in the case of the former, we correct the eigenfrequency from the obtained perturbations by determining the curvature from fitting. This is based on the harmonic approximation of the potential energy surface E_{harm} at the point x_0, that is either an obtained transition state or a minimum. $\overleftrightarrow{H}$ is the Hessian and $\overleftrightarrow{M}$ the reduced mass matrix. For small deviations along an eigenmode j ($x_0 + a\vec{u}_{\mathrm{j}}$), this reduces to:

$$E_{\mathrm{harm}}\left(x_0 + a\vec{u}_{\mathrm{j}}\right) = E(x_0) + \frac{1}{2}a^2\vec{u}_{\mathrm{j}}^{\,T}\overleftrightarrow{H}\,\vec{u}_{\mathrm{j}} \qquad \text{(Equation 2)}$$

$$E_{\mathrm{harm}}\left(x_0 + a\vec{u}_{\mathrm{j}}\right) = E(x_0) + \frac{1}{2}a^2\vec{u}_{\mathrm{j}}^{\,T}\overleftrightarrow{M}^{-1}(\overleftrightarrow{M}\overleftrightarrow{H})\,\vec{u}_{\mathrm{j}} \qquad \text{(Equation 3)}$$

$$E_{\mathrm{harm}}\left(x_0 + a\vec{u}_{\mathrm{j}}\right) = E(x_0) + \frac{1}{2}a^2\vec{u}_{\mathrm{j}}^{\,T}\overleftrightarrow{M}^{-1}\omega_{\mathrm{j}}^2\,\vec{u}_{\mathrm{j}} \qquad \text{(Equation 4)}$$

$$E_{\mathrm{harm}}(a) = E(x_0) + \frac{1}{2}c_{\mathrm{j}}a^2 \qquad \text{with } c_{\mathrm{j}} = \omega_{\mathrm{j}}^2 m_{\mathrm{j}} \text{ and } m_{\mathrm{j}} = \vec{u}_{\mathrm{j}}^{\,T}\overleftrightarrow{M}^{-1}\,\vec{u}_{\mathrm{j}} \qquad \text{(Equation 5)}$$

This now enables translating the curvature $c_{\mathrm{j,fit}}$ of the perturbed data (force constant) into a vibration frequency via $\omega_{\mathrm{j,fit}} = \sqrt{c_{\mathrm{j,fit}}/m_{\mathrm{j}}} = 2\pi\nu_{\mathrm{j,fit}}$.

Figure S10 visualizes the results of this approach for all instable modes of minima and transition states. Three of the calculated transition states exhibit an additional instable mode. Also, for three of the minima one instable frequency was found. But when comparing the curvatures from the vibrational analyses with the distinct perturbations along the instable modes, we identify all additional instable vibration frequencies to result from numerical inaccuracies. By harmonically fitting the energies of the perturbed points all obtained vibration frequencies were corrected.

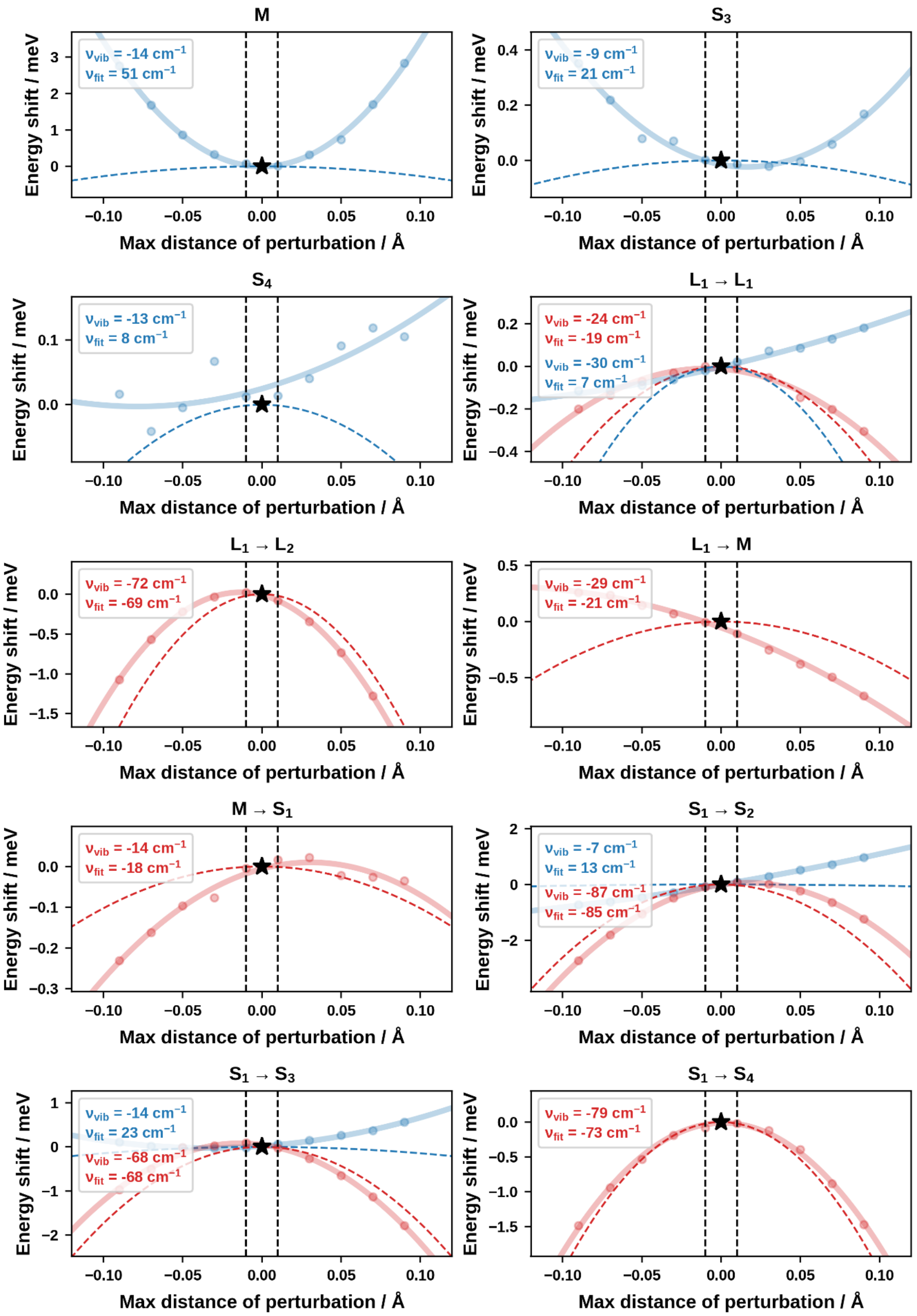

Figure S10: *Displacements along all instable vibration modes appearing at minima and states. A schematic description of the content is provided in Figure S9.*

5.2.3 Uncertainty estimation of vibrational frequencies

The uncertainty estimation of the computed vibrational frequencies depends also on the quality of the obtained extremum. For the case that the computed extremum lies within the harmonic range of the real extremum, the curvatures and vibration frequencies should be constant. In practice, numerical instabilities, which are caused by DFT, can additionally distort vibration frequencies (for details see Section 5.2.2).

To get a rough estimate of the uncertainty of vibrational frequencies, the process described in the previous section was repeated for six eigenmodes with frequencies from 11 to 2218 cm^{-1}, as displayed in Figure S11. This was done for the rate-limiting transition state of the joint process of standing up ($L_1 \rightarrow S_1$).

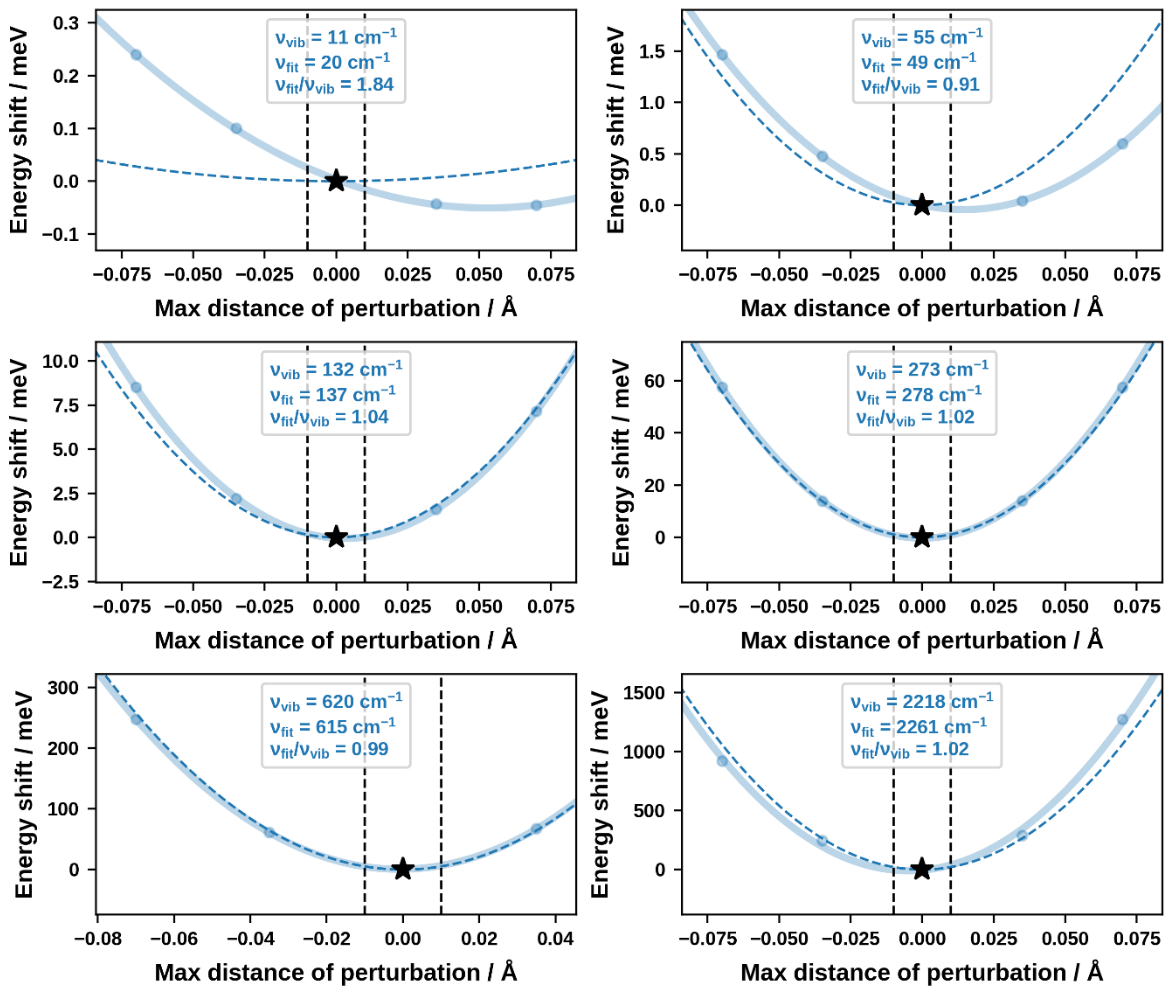

Figure S11: *Perturbations along different vibration modes of the rate-limiting transition state of $L_1 \rightarrow S_1$. A schematic description of the content is provided in Figure S9.*

5.3 Attempt frequencies

The attempt frequency A is obtained via the stable frequencies of the initial (ν_i^{ini}) and the transition state (ν_i^{TS}), as stated in Equation 6. The ansatz and the result of the relative uncertainty are given by Equation 7 and 8, respectively.

$$A = \frac{\prod_{i=1}^{3N} \nu_i^{ini}}{\prod_{i=1}^{3N-1} \nu_i^{TS}} = \frac{P^{ini}}{P^{TS}} \qquad \text{with } P = \prod_i \nu_i \qquad \text{(Equation 6)}$$

$$\Delta A = \sqrt{\left(\frac{\partial A}{\partial P^{ini}}\right)^2 \cdot (\Delta P^{ini})^2 + \left(\frac{\partial A}{\partial P^{TS}}\right)^2 \cdot (\Delta P^{TS})^2} \qquad \text{with } \frac{\Delta P}{P} = \sqrt{\sum_i \left(\frac{\Delta \nu_i}{\nu_i}\right)^2} \qquad \text{(Equation 7)}$$

$$\frac{\Delta A}{A} = \sqrt{\sum_{i=1}^{3N} \left(\frac{\Delta \nu_i^{ini}}{\nu_i^{ini}}\right)^2 + \sum_{i=1}^{3N-1} \left(\frac{\Delta \nu_i^{TS}}{\nu_i^{TS}}\right)^2} \qquad \text{(Equation 8)}$$

The relative uncertainty of the attempt frequency solely depends on the relative uncertainties of the computed vibration frequencies. Based on the results of Figure S11 and the influence of conducting the vibrational analysis for the results of the 4x4 super cell without substrate relaxation, we estimate the relative uncertainty of the attempt frequencies to $\frac{\Delta A}{A} \approx 10$.

5.4 Suppression temperatures

In the main manuscript temperatures T_{supp} are proposed that sufficiently suppress transitions of individual molecules. In detail, the temperatures T_{supp} are determined for transition rates of k_{supp} = 1 day^{-1} via Equation 9 (energy barrier $\Delta E^{\ddagger}$, the attempt frequency A).

$$T_{supp} = \frac{\Delta E^{\ddagger}}{k_B \left(\ln (A) - \ln (k_{supp})\right)} \qquad \text{(Equation 9)}$$

To get a rough feeling for the uncertainties of the suppression temperatures we perform a simple error propagation. The general approach and the resulting description are shown in Equations 10 and 11, respectively.

$$\Delta T_{supp} = \sqrt{\left(\frac{\partial T_{supp}}{\partial \Delta E^{\ddagger}}\right)^2 \cdot (\Delta\Delta E^{\ddagger})^2 + \left(\frac{\partial T_{supp}}{\partial A}\right)^2 \cdot (\Delta A)^2 + \left(\frac{\partial T_{supp}}{\partial k_{supp}}\right)^2 \cdot \left(\Delta k_{supp}\right)^2} \qquad \text{(Equation 10)}$$

$$\Delta T_{supp} = T_{supp} \cdot \sqrt{\left(\frac{\Delta\Delta E^{\ddagger}}{\Delta E^{\ddagger}}\right)^2 + \left(\frac{1}{\ln(A) - \ln(k_{supp})} \cdot \frac{\Delta A}{A}\right)^2 + \left(\frac{1}{\ln(A) - \ln(k_{supp})} \cdot \frac{\Delta k_{supp}}{k_{supp}}\right)^2} \qquad \text{(Equation 11)}$$

In Equation 11, it is evident that the uncertainty of the suppression temperatures grows with its nominal value. For all transitions $\Delta\Delta E^{\ddagger}$ = 0.06 eV is assumed, which corresponds approximately to the double of the uncertainty of the adsorption energies obtained via DFT, and a relative error of one order of magnitude for A, i.e. $\frac{\Delta A}{A}$ = 10 (for more details see Section 5.3). Δk_{supp} is not accounted for (Δk_{supp} = 0).

The results for the forward and reverse transitions are visualized in Figure S12 and stated in Table S5 and S6, respectively. Therein, the nominal values (T_{supp}) and the uncertainty estimate (ΔT_{supp}) of the suppression temperature are stated, as well as the single uncertainty contributions regarding the attempt frequency and the energy barrier.

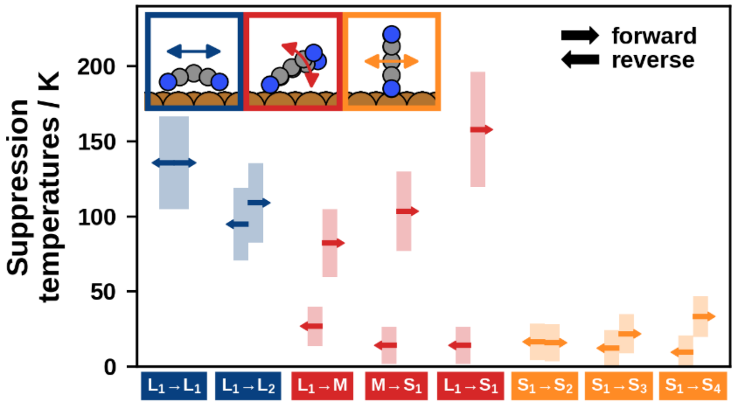

Figure S12: *Estimated temperatures for suppression obtained for transition rates of 1 day^{-1}. The arrow directions indicate the suppression temperatures required for forward or reverse transitions. Shaded areas indicate the temperature uncertainties connected to the respective transition.*

Table S5: *Uncertainties of suppression temperatures of forward transitions*

Transition	T_{supp} / K	ΔT_{supp} / K	$\left\lvert\frac{\partial T_{supp}}{\partial A}\cdot\Delta A\right\rvert$ / K	$\left\lvert\frac{\partial T_{supp}}{\partial \Delta E^{\ddagger}}\cdot\Delta\Delta E^{\ddagger}\right\rvert$ / K
$L_1 \rightarrow L_1$	136	33	29	15
$L_1 \rightarrow L_2$	109	29	24	16
$L_1 \rightarrow M$	82	26	20	17
$M \rightarrow S_1$	103	29	24	16
$L_1 \rightarrow S_1$	158	40	36	16
$S_1 \rightarrow S_2$	16	18	4	18
$S_1 \rightarrow S_3$	22	19	5	18
$S_1 \rightarrow S_4$	33	18	8	17

Table S6: *Uncertainties of suppression temperatures of reverse transitions*

Transition	T_{supp} / K	ΔT_{supp} / K	$\left\lvert\frac{\partial T_{supp}}{\partial A}\cdot\Delta A\right\rvert$ / K	$\left\lvert\frac{\partial T_{supp}}{\partial \Delta E^{\ddagger}}\cdot\Delta\Delta E^{\ddagger}\right\rvert$ / K
$L_1 \rightarrow L_1$	136	33	29	15
$L_2 \rightarrow L_1$	95	27	22	16
$M \rightarrow L_1$	27	18	7	17
$S_1 \rightarrow M$	14	18	4	18
$S_1 \rightarrow L_1$	14	18	4	18
$S_2 \rightarrow S_1$	17	18	4	17
$S_3 \rightarrow S_1$	12	18	3	18
$S_4 \rightarrow S_1$	10	17	2	17

6 References

(1) Egger, A. T.; Hörmann, L.; Jeindl, A.; Scherbela, M.; Obersteiner, V.; Todorović, M.; Rinke, P.; Hofmann, O. T. Charge Transfer into Organic Thin Films: A Deeper Insight through Machine-Learning-Assisted Structure Search. *Adv. Sci.* **2020**, *7* (15), 2000992. https://doi.org/10.1002/advs.202000992.

(2) Reuter, K.; Scheffler, M. Composition, Structure, and Stability of RuO2(110) as a Function of Oxygen Pressure. *Phys. Rev. B* **2001**, *65* (3), 035406. https://doi.org/10.1103/PhysRevB.65.035406.

(3) Hjorth Larsen, A.; JØrgen Mortensen, J.; Blomqvist, J.; Castelli, I. E.; Christensen, R.; Dułak, M.; Friis, J.; Groves, M. N.; Hammer, B.; Hargus, C.; Hermes, E. D.; Jennings, P. C.; Bjerre Jensen, P.; Kermode, J.; Kitchin, J. R.; Leonhard Kolsbjerg, E.; Kubal, J.; Kaasbjerg, K.; Lysgaard, S.; Bergmann Maronsson, J.; Maxson, T.; Olsen, T.; Pastewka, L.; Peterson, A.; Rostgaard, C.; SchiØtz, J.; Schütt, O.; Strange, M.; Thygesen, K. S.; Vegge, T.; Vilhelmsen, L.; Walter, M.; Zeng, Z.; Jacobsen, K. W. The Atomic Simulation Environment - A Python Library for Working with Atoms. *Journal of Physics Condensed Matter*. Institute of Physics Publishing June 7, 2017, p 30. https://doi.org/10.1088/1361-648X/aa680e.

(4) Ochterski, J. W. *Vibrational Analysis in Gaussian*.
